# Homologous recombination-dependent repair of telomeric DSBs in proliferating human cells

**DOI:** 10.1038/ncomms12154

**Published:** 2016-07-11

**Authors:** Pingsu Mao, Jingfan Liu, Zepeng Zhang, Hong Zhang, Haiying Liu, Song Gao, Yikang S. Rong, Yong Zhao

**Affiliations:** 1 Key Laboratory of Gene Engineering of the Ministry of Education, Department of Biochemistry, School of Life Sciences, Sun Yat-sen University, Guangzhou 510006, China; 2 Collaborative Innovation Center of High Performance Computing, National University of Defense Technology, Changsha 410073, China; 3 Zhongshan Medical School, Sun Yat-sen University, Guangzhou 510006, China; 4 State Key Laboratory of Oncology in South China, Collaborative Innovation Center for Cancer Medicine, Sun Yat-sen University Cancer Center, Guangzhou 510060, China

## Abstract

Telomeres prevent chromosome ends from being recognized as double-stranded breaks (DSBs). Meanwhile, G/C-rich repetitive telomeric DNA is susceptible to attack by DNA-damaging agents. How cells balance the need to protect DNA ends and the need to repair DNA lesions in telomeres is unknown. Here we show that telomeric DSBs are efficiently repaired in proliferating cells, but are irreparable in stress-induced and replicatively senescent cells. Using the CRISPR-Cas9 technique, we specifically induce DSBs at telomeric or subtelomeric regions. We find that DSB repair (DSBR) at subtelomeres occurs in an error-prone manner resulting in small deletions, suggestive of NHEJ. However, DSBR in telomeres involves ‘telomere-clustering', 3′-protruding C-rich telomeric ssDNA, and HR between sister-chromatid or interchromosomal telomeres. DSBR in telomeres is suppressed by deletion or inhibition of Rad51. These findings reveal proliferation-dependent DSBR in telomeres and suggest that telomeric HR, which is normally constitutively suppressed, is activated in the context of DSBR.

## Abstract (web summary)

Telomeres protect the ends of chromosomes from the DNA damage recognition machinery, however how damage in telomeres is repaired is poorly understood. Here the authors use CRISPR-Cas9 to induce DNA breaks and identify proliferation dependent homologous recombination repair.

## Graphical abstract

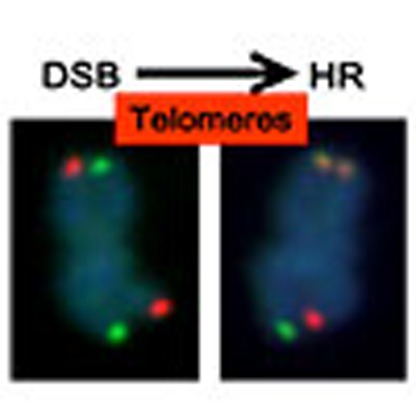

Human telomeres are composed of tandem repeats of the DNA sequence TTAGGG/AATCCC and a complex of proteins called shelterin, which protects chromosome ends from attrition, degradation, promiscuous recombinogenic events and end-to-end ligations that result in fusion with other chromosomes^1^,^2^,^3^. Telomeric DNA terminates with 3′ single-stranded G-rich overhangs that can be inserted into homologous double-stranded regions, resulting in a lasso-like telomere loop (t-loop) structure thought to prevent chromosome ends from being recognized as double-stranded breaks (DSBs)^4^.

The requirement to protect chromosome ends must be balanced with the need to repair DNA damage that occurs in telomere regions. At an estimate, human cells accumulate ∼10 (ref. 5) spontaneous DNA lesions per cell per day^5^,^6^. Because the guanine nucleotide is especially susceptible to oxidative attack, the G-rich strand of telomeric DNA is particularly sensitive to damage from ultraviolet light and other oxidative DNA damaging agents^7^,^8^. Some studies suggest that DNA lesions may be repaired less efficiently in telomeres than in the rest of the genome^7^,^9^, possibly due to the heterochromatic nature of telomeric chromatin^10^ and/or inhibition of non-homologous end-joining (NHEJ) by telomeric-repeat binding factor 2 (TRF2)^11^,^12^,^13^. However, many details of telomeric DNA lesion repair remain unclear. Whereas a previous study suggested that telomeric DNA damage is resistant to repair^14^, another study showed that telomeric DSBs are repaired within 48 h (ref. 15). Such conflicting results could be explained by the use of different experimental methods (that is, DNA lesions induced with different agents or in a different manner), or by the initiation of cell senescence when the amount of DNA damage becomes too high^16^,^17^. Importantly, previous studies did not directly examine whether the proliferative state of the cell affects the fate of telomeric DNA damage.

The ability to repair DNA lesions is critical for cell viability. A persistent DSB induces a potent DNA damage response (DDR) leading to cell cycle arrest, cell senescence or apoptosis that ultimately results in lethality at the cellular level^18^. DSB repair (DSBR) has at least two pathways: the error-prone non-homologous end joining (NHEJ) pathway and the error-free homologous recombination (HR) pathway^19^,^20^. NHEJ involves minimal processing of the damaged DNA by nucleases, followed by direct re-ligation of the DNA ends. NHEJ introduces small deletions into the genome, and is therefore intrinsically mutagenic. By contrast, HR proceeds through a ssDNA intermediate, and requires a homologous DNA template, usually the intact sister chromatid, but allows for error-free non-mutagenic repair of the DSB^21^.

TRF2, which is bound to telomere ends, suppresses NHEJ and prevents end fusion between telomeres. Because of the repetitive nature of telomeric DNA, it was believed that HR is also generally suppressed in telomeres^22^. However, some evidence suggests an active role for HR at telomeres. For example, telomeric HR is activated in human alternative lengthening of telomeres (ALT) cancer cells^22^ and has been shown to function in telomere maintenance in response to DSBs in telomeres^23^. Moreover, protein factors known to play a role in HR are associated with telomeres in a cell cycle-dependent manner^24^. In particular, depletion of Rad51d, a key factor in HR, results in telomere shortening and chromosome instability in mouse cells^25^. These results suggest that HR may play a role in normal telomere maintenance.

The subtelomeric region is larger than the telomeric region of the chromosome, and is typically composed of various repeated elements, pseudogenes and retrotransposons^26^. Previous studies have not carefully distinguished the effects of DNA damage in the telomeric region of the chromosome from the effects of DNA damage in subtelomeric regions. Here we generated DSBs in subtelomeric or telomeric DNA sequences and followed their fate in different human cell types. Our results show that telomeric DSBs are efficiently repaired in proliferating human cells, including normal and cancer cells, but are inefficiently repaired in senescent human cells with persistent DDR. Subtelomeric DSBs are repaired in an error-prone manner resulting in small deletions, suggesting a mechanism involving NHEJ. In contrast, multiple features of DSB repair in telomeric DNA points to the involvement of homologous recombination (HR) between sister and non-sister chromatids. The implications of these results are discussed.

## Results

### Repair of telomeric DSBs in human fibroblasts and HeLa cells

**Figure 1:**
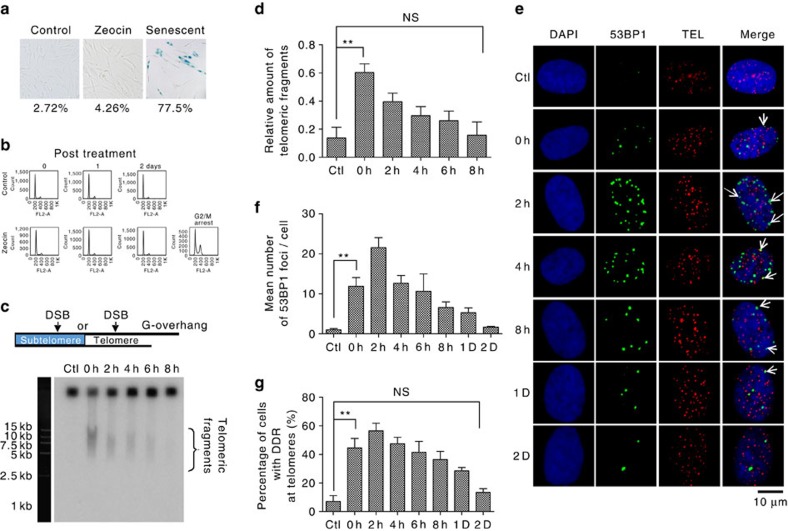
Repair of telomeric DSBs in BJ fibroblast cells. (**a**) Cells were treated with zeocin (100 μg ml^−1^) for 1 h. Cells were stained to detect SA-β-gal activity before (Control) and 24 h after treatment (Zeocin). Senescent BJ fibroblast cells were used as a positive staining control. (**b**) Zeocin treated (100 μg ml^−1^ for 1 h) and untreated cells (Control) were subjected to cell cycle FACS analysis. Stress-induced senescent cells were used as a positive control for G2/M arrest. (**c**) Cells were exposed to zeocin (100 μg ml^−1^) for 1 h and then given fresh medium for 0, 2, 4, 6 and 8 h. Cells were collected at the indicated time points and analysed by constant-field gel electrophoresis (CFGE). Telomeric fragments were detected by hybridization of their G-rich overhangs with C-rich probe under native conditions. Corresponding MW was indicated on the left. (**d**) Quantification of **c**. The relative amount of G-rich overhang on telomeric fragments was determined as the signal intensity of the smear normalized to the intensity in the entire sample. Untreated cells served as a control (Ctl). (**e**) Cells were exposed to zeocin (100 μg ml^−1^) for 1 h and then given fresh medium for 0, 2, 4, 6, 8, 24 and 48 h. Untreated cells served as a control (Ctl). DDR and telomeres were visualized using antibody against 53BP1 (IF) or a probe to a telomeric sequence (FISH), respectively. Arrows indicate merged foci. Scale bar, 10 μm. (**f**) Quantification of **e**. The mean number of 53BP1 foci per cell was determined. (**g**) Quantification of **e**. The number of cells with 1 or more 53BP1 foci coincident with the signal from the telomere probe was counted, and the percentage of cells with 1 or more telomeric 53BP1 foci per cell was calculated. All values are average ±s.d. of three independent experiments (*n*≥100). ^**^P<0.01. The Student's *t*-test was used to determine the statistical significance.

To explore the fate of telomeric DNA lesions, we treated cultured cells with zeocin. Zeocin is a radio-mimetic chemical that induces several types of DNA damage (for example, oxidative, single- and double-stranded breaks) in cultured cells^27^, and has been widely used to induce random DSBs at the genome^28^,^29^. We treated normal human BJ fibroblasts with 100 μg ml^−1^ zeocin for 1 h, conditions under which no senescent (Fig. 1a) or arrested cells were observed (Fig. 1b). After treatment with zeocin, the media was exchanged for zeocin-free medium and cells were collected at 2 h time intervals (0, 2, 4, 6 and 8 h). Cells were embedded in agarose, lysed *in situ*, and nucleic acids were subjected to constant-field gel electrophoresis (CFGE). Intact genomic DNA does not enter the gel, and the amount of DNA released into the gel as linear DNA fragments is proportional to the number of DSBs in the genome at the time of harvesting. Electrophoresis was followed by hybridization with a C-rich telomere-specific probe under native conditions^30^. Nondenaturing hybridization with the C-rich probe identifies the fraction of telomeric fragments carrying a single-stranded G-rich overhang at the end of the chromosome, and allows the relative amount of telomeric overhangs in the fragments resulting from the double-strand breaks in telomeric and sub-telomeric DNA to be determined. Immediately after treatment with zeocin, a smear of telomere-homologous DNA fragments of variable length was detected (Fig. 1c). The abundance of these fragments, as quantified by the relative amount of their G-rich overhangs, decreases to background level within 8 h of zeocin treatment (Fig. 1c,d).

We also visualized DSBs in cells by immuno-staining and fluorescence *in situ* hybridization (IF-FISH), using antibody to 53BP1 and a telomere-specific DNA hybridization probe (Fig. 1e). In spite of another possibly minor role for 53BP1 other than in DDR^31^,^32^, 53BP1 has been widely used as a marker for DSBs. We calculated the number of 53BP1 foci per nucleus as well as the fraction of telomeric DNA sequences in 53BP1 foci. As expected, the number of 53BP1 foci increased to an average of ∼21 foci per nucleus 2 h after the release from treatment, and the percentage of cells with telomeric 53BP1 foci (53BP1 foci colocalized with telomeres) increased accordingly (Fig. 1f,g). However, both values decreased slowly and returned to background levels within 2 days of zeocin treatment (Fig. 1f,g). We obtained similar results when BJ fibroblast cells were exposed to 2 Gy of ionizing irradiation (Supplementary Fig. 1).

Taken together, our data support the conclusion that telomeric DSBs can be repaired in BJ fibroblasts, although the kinetics of DSBR appeared to differ depending on whether DSBs were quantified by CFGE or IF-FISH (8 h versus 2 days, respectively). We also observed the rapid repair of telomeric DSBs in HeLa cells (a human cervical carcinoma-derived cell line) (Supplementary Fig. 2). Again, the repair kinetics differed, with telomeric fragments detected by CFGE decreasing to background level in 2 h and telomeric 53BP1 foci detected by IF-FISH decreasing to background in 8 h (Supplementary Fig. 2). Compared with BJ fibroblasts, the faster kinetics of DSBR in HeLa cells may reflect a shorter cell cycle (20 versus 48 h for BJ fibroblasts) and a faster proliferation rate.

### Repair of telomeric DSBs in senescent cells

**Figure 2:**
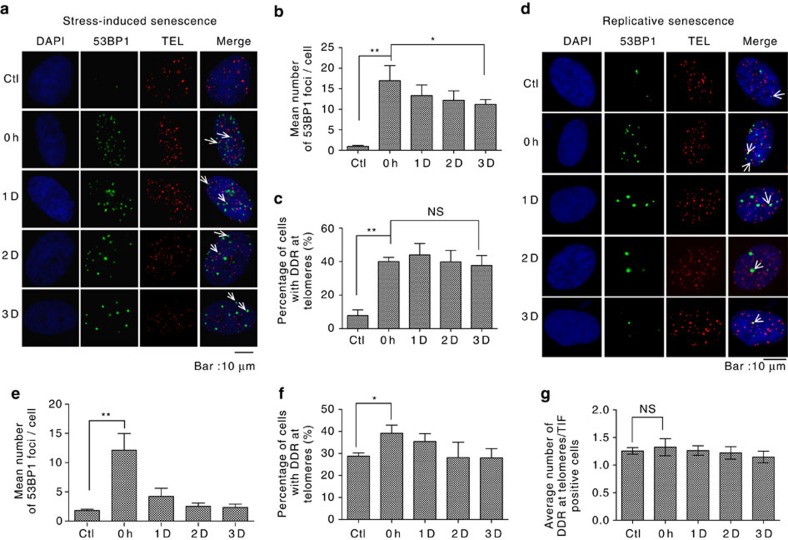
Repair of telomeric DSBs in senescent cells. (**a**) Cells were treated with zeocin (100 μg ml^−1^) for 48 h and then given fresh medium for 1, 2 or 3 days. DDR and telomeres were visualized using antibody against 53BP1 (IF) or a probe to a telomeric sequence (FISH), respectively. Arrows indicate merged foci. Scale bar, 10 μm. (**b**) Quantification of **a**. The mean number of 53BP1 foci per cell was determined. (**c**) Quantification of **a**. The percentage of cells with one or more telomeric 53BP1 foci per cell was determined. (**d**) Same as in **a**, except cells that senesced due to replicative exhaustion were used. Arrows indicate merged foci. Scale bar, 10 μm. (**e**) Quantification of **d**. The mean number of 53BP1 foci per cell was determined. (**f**) Quantification of **d**. The percentage of cells with one or more telomeric 53BP1 foci per cell was determined. (**g**) Quantification of **d**. Average number of telomeric 53BP1 foci in telomere dysfunction induced foci (TIF) positive cells was determined before (Ctl) and after treatment (0, 1, 2 and 3 days). All values are the average ±s.d. of three independent experiments (*n*≥100). *P<0.05; ^**^P<0.01. The Student's *t*-test was used to determine the statistical significance.

A phenomenon known as stress-induced senescence is induced when the number of DNA lesions in a cell exceeds the capacity of the cell to repair the lesions^33^. In a previous study, cells exposed to high-dose irradiation (for example, 10 or 20 Gy) were reported to have persistent telomeric DSBs and DDR. Because high-dose radiation can induce senescence^14^,^33^,^34^, we postulated that the failure to repair telomeric DSBs observed in the previous studies might correlate with cell senescence. To test this idea, we treated human BJ fibroblast cells with 100 μg per ml zeocin for 48 h, after which 76% of cells were senescent, as indicated by positive staining for SA-β-gal (Supplementary Fig. 3a). After removal of the drug, we monitored DSBR by detection of 53BP1 foci. Zeocin treatment caused an increase in the numbers of total and telomeric 53BP1 foci (Fig. 2a–c). Interestingly, the number of 53BP1 foci/nucleus decreased gradually over time (Fig. 2b), but the number of telomeric 53BP1 foci/nucleus did not change over time (Fig. 2c). However, we did not observe telomere fusion (that is, as would be indicated by a high molecular weight signal on a TRF gel)^11^, or a decrease in the abundance of telomeric overhangs (Supplementary Fig. 4a), excluding the possibility that persistent telomeric 53BP1 foci could be caused by the ‘telomere uncapping' often associated with telomere fusion and degradation of telomeric overhangs^11^,^35^,^36^. To address the possibility that a bulk cell assay may not be sensitive enough to detect a few uncapped telomeres in the entire cell population, we determined the relative length of 53BP1 occupied telomeres (telomeres with colocalized 53BP1 foci) by IF-FISH. Our results showed that 53BP1 foci are not preferentially localized to critically short telomeres (uncapped telomeres) (Supplementary Fig. 4b). Taken together, these data demonstrate that persistent telomeric DSBs are associated with stress-induced senescence.

We also monitored DSBR in replicatively senescent cells for comparison with our results from stress-induced cells. Human BJ fibroblasts were cultured *in vitro* for ∼72 population doublings (PDs) until they senesced due to replicative exhaustion, as indicated by positive staining for SA-β-gal in 72% of the cells (Supplementary Fig. 3b). DSBR was monitored in these cells after treatment with zeocin (100 μg ml^−1^) for 1 h, which generated reparable 53BP1 foci-associated DSBs in bulk chromatin (Fig. 2d,e). Repair was relatively efficient, with return to background level within 2 days (Fig. 2d,e). However, in replicatively senescent cells, telomere dysfunction-induced foci (TIFs) were observed before and after treatment with zeocin (Fig. 2d,f), and very few new telomeric 53BP1 foci were induced by zeocin (Fig. 2f). To further explore newly formed telomeric 53BP foci in replicatively senescent cells following zeocin treatment, we selectively sorted for cells with telomeric 53BP1 foci (TIF positive cells) and determined the average number of telomeric 53BP1 foci per cell before and after zeocin treatment. We found that newly formed foci were not induced in the replicatively senescent cells (Fig. 2g). Our interpretation of this result is that telomeric DNA in replicatively senescent cells may be insensitive to zeocin, or alternatively, that the more abundant telomeric heterochromatin present in aged cells versus young cells^37^ prevents telomeric DSBs from eliciting DDR.

### DSBR in subtelomeric DNA is mediated by NHEJ

**Figure 3:**
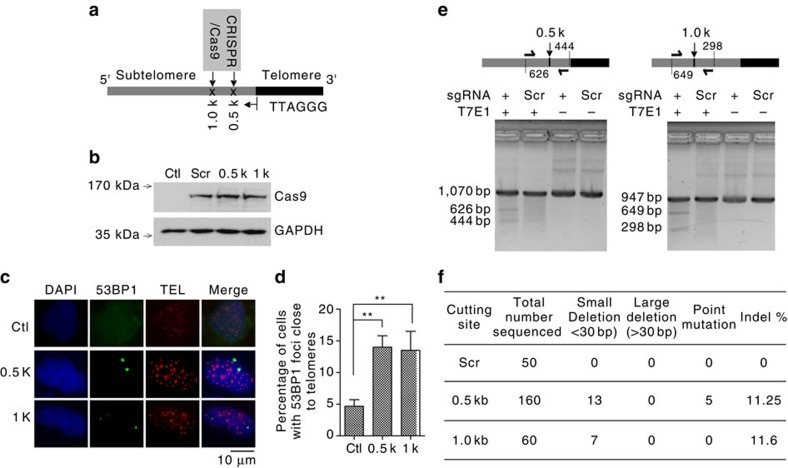
Repair of CRISPR-Cas9-induced DSBs in the subtelomeric region. (**a**) Schematic diagram showing the strategy used to induce DSBs in the subtelomeric region (0.5 and 1 kb from the first TTAGGG sequence in Xp/Yp). (**b**) Western blot analysis of Cas9 expression. Non-transfected cells served as a transfection control (Ctl) and plasmid with scrambled sgRNA (Scr) was used as a control for induction of DSBs. (**c**) Representative images of DSB repair foci in the subtelomere region. Scale bar, 10 μm. (**d**) Quantification of **c**. All values are the average ±s.d. of three independent experiments (*n*=200). ^**^P<0.01. The Student's *t*-test was used to determine the statistical significance. (**e**) T7 endonuclease I (T7E1) susceptibility was used to screen and identify mutations and deletions introduced during DSBR. Cells exposed to scrambled sgRNA (Scr) were used as a control. The location of PCR primer hybridization, ∼1 kb apart, is shown. (**f**) A summary of the results from sequencing the PCR products.

Ionizing radiation and zeocin are relatively non-specific DNA-damaging agents that induce DSBs throughout the genome, including the telomeric and subtelomeric regions. To specifically analyse DSBR in subtelomeric DNA sequences, we used the CRISPR-Cas9 technique to introduce a DSB 0.5 or 1 kb from the first TTAGGG on human Xp/Yp chromosome arms (Fig. 3a)^38^. We transfected a plasmid carrying the Cas9 gene and the targeting sgRNA was transfected into human 293T cells, and confirmed the expression of Cas9 by western blot (Fig. 3b). 53BP1 foci close to telomere-hybridizing DNA (IF-FISH) was observed in ∼15% of the cells (Fig. 3c,d). The low efficiency of target site cleavage by Cas9 may reflect the high content of heterochromatin in the subtelomeric region^10^.

We next investigated the DNA sequence at the repaired Cas9-mediated subtelomeric DSBs by screening for partial or complete homology to the original cutting site using T7 endonuclease I enzyme (T7E1 assay)^39^, an enzyme that cleaves heteroduplex dsDNA but does not cleave homoduplex dsDNA. Our results showed 13% or 15% sensitivity to T7E1 for target cleavage sites 0.5 or 1.0 kb into the subtelomeric region (Fig. 3e), respectively, suggesting that DSBR in subtelomeric DNA is error-prone, and likely mediated by NHEJ. We confirmed this result by sequencing cloned fragments corresponding to a 1-kb region surrounding the repaired subtelomeric DSBs. We found that ∼11% of the clones differed in sequence from the cleavage site, and that a majority (20 out of 25) of the mutant clones carried small deletions, but none of the mutants contained large deletions (Fig. 3f, Supplementary Fig. 5 for details). This supports the conclusion that subtelomeric DSBs are repaired by classical NHEJ (C-NHEJ) rather than microhomology-mediated end joining.

### Telomeric DSBs lead to no telomere loss or cell senescence

**Figure 4:**
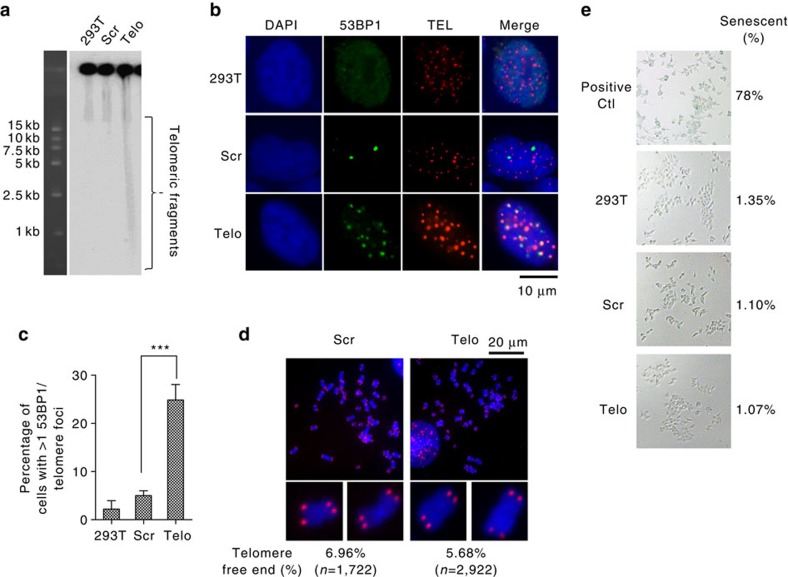
Repair of CRISPR-Cas9-induced telomeric DSBs in 293T cells. (**a**) DSBs were induced at telomeres by expression of telomeric sgRNA (Telo) in CRISPR-Cas9-transfected 293T cells. Scrambled sgRNA (Scr) was used as a control. DNA was isolated and analysed by CFGE as in Fig. 1c. (**b**) Immunofluorescence and FISH analysis was performed 24 h after CRISPR-Cas9 transfection of 293T cells. Normal 293T cells and cells exposed to scrambled sgRNA (Scr) were used as controls. Scale bar, 10 μm.(**c**) Quantification of **b**. The percentage of cells with one or more telomeric 53BP1 foci per cell was calculated. Values are the average ±s.d. of three independent experiments (*n*≥100). ^***^P<0.001. The Student's *t*-test was used to determine the statistical significance. (**d**) Telomere signal at chromosome termini determined using FISH. The percentage of chromosomes with one or more telomere-free ends was calculated, based on *n*=1,722 or 2,922 total chromosomes for Scr or Telo-sgRNA samples, respectively. Scale bar, 20 μm. (**e**) Cells treated as in **b** were analysed for SA-β-gal activity. Stress-induced 293T senescent cells were used as a positive staining control. Approximately 1,000 cells were counted and the percentage of senescent cells was calculated.

In a previous study, the CRISPR-Cas9 system was successfully used to target telomeric DNA sequences in human cells^40^. Here we used a similar approach to generate telomeric DSBs in 293T-derived cells, which were then analysed by CFGE and IF-FISH to evaluate cleavage efficiency and DDR status. Our CFGE data revealed telomere-homologous DNA fragments of variable size in CRISPR-Cas9 modified 293T cells, but not in wild-type 293T cells or when a non-specific sgRNA was used (for example, scrambled control) (Fig. 4a). IF-FISH data revealed that 25% of the cells had one or more telomeric 53BP1 foci (Fig. 4b,c). In these cells ∼60% of 53BP1 foci were localized to telomeres. The remaining non-telomeric 53BP1 foci may have resulted from non-specific cleavage by Cas9, the presence of short interstitial TTAGGG sequence, or activation of DDR at chromosome ends following complete telomere loss^41^,^42^. We addressed whether the CRISPR-Cas9 caused significant telomere loss by performing FISH and quantifying telomere-free ends. However, we found that the CRISPR-Cas9 modified 293T cells did not accumulate telomere-free ends (Fig. 4d). This is consistent with the idea that induced DSBs in CRISPR-Cas9 modified 293T cells activate DDR and are likely to be repaired.

A previous study by Fumagalli *et al.*^14^ reported that telomeric DNA damage, including DSBs, remain unrepaired and lead to persistent DDR and senescence. To test whether induction of DSBs at telomeres induces senescence, we identified senescent cells in the CRISPR-Cas9 modified 293T cell population by SA-β-gal staining. Based on the fraction of SA-β-gal positive-staining cells, we concluded that the fraction of senescent cells is the same in CRISPR-Cas9 modified 293T cells with telomere-targeted DSBs as in control CRISPR-Cas9 modified 293T cells treated with scrambled RNA or in the control parental 293T cells (Fig. 4e). In addition, CRISPR-Cas9 modified 293T cells with telomere-targeted DSBs showed no increase in cell cycle arrest or apoptosis (Supplementary Fig. 6a,b).

### Activation of HR by telomeric DSBs

**Figure 5:**
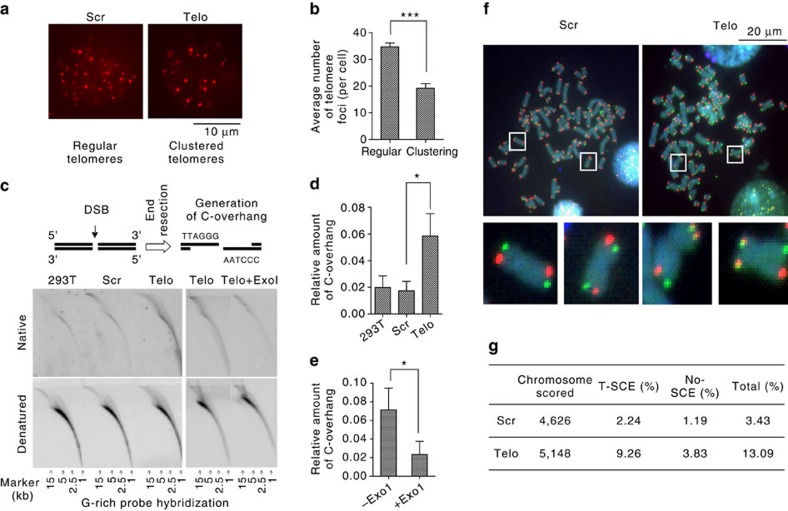
Evidence for HR-mediated repair of telomeric DSBs. (**a**) Cells were prepared as described in Fig. 4b. Telomeres were visualized using FISH. A representative image shows large foci, indicating clustering of telomeres in cells with telomeric DSBs. Scrambled sgRNA was used as a control. Scale bar, 10 μm (**b**) Quantification of **a**, showing the average number of telomere foci in CRISPR-Cas9-targeted cells. Values are the average ±s.d. of three independent experiments (*n*=100). ^***^P<0.001. (**c**) Cells were prepared as in Fig. 4a. DNA was prepared and analysed by 2D agarose gel electrophoresis, followed by hybridization with a G-rich telomere strand-specific probe under native or denatured conditions. ExoI treatment of DNA prior to 2D gel electrophoresis eliminated the signal from ss-C-rich DNA. (**d**) Quantification of the C-overhang. Values are the average ±s.d. of three independent experiments. *P<0.05. (**e**) Quantification of the C-overhang sensitive to ExoI digestion. Values are the average ±s.d. of three independent experiments. *P<0.05. (**f**) Detection of T-SCE by CO-FISH (see methods). Yellow dots indicate the presence of T-SCE. Cells were transfected with telomeric sequence (Telo) or scrambled sgRNA (Scr) as indicated. Scale bar, 20 μm. (**g**) Summary of CO-FISH results. The Student's *t*-test was used to determine the statistical significance.

In general, telomere clustering occurs only in meiotic cells^43^. However, after telomeric DSBs were induced in CRISPR-Cas9 modified 293T cells, we noted that approximately one-third (31.3%) of the cells displayed unusually large foci, suggesting that multiple telomeres were in physical proximity to each other in a ‘cluster'. These clustered foci were much less common (3.4%) in control cells treated with scrambled sgRNA (Fig. 5a). Accordingly, the number of telomeric foci per nucleus decreased significantly from ∼35 to ∼19 foci (Fig. 5b). Large telomeric foci in human ALT cells were previously attributed to a cluster of telomeres, and in this context, it was proposed that telomeric DSBs trigger long-range movement of chromosomes and clustering of chromosome ends, leading to homology-directed telomere synthesis^23^.

We tested the idea that telomeric DSBs can be repaired by an HR-dependent process by looking for intermediates of HR-dependent repair and/or the direct consequence of HR, namely an increased frequency of telomeric sister chromatid exchange (T-SCE). First, cells were lysed and genomic DNA was analysed by two-dimensional (2D)-gel electrophoresis followed by non-denaturing hybridization with a G-rich probe that detects telomeric 3′ C-rich ssDNA, an intermediate of HR-mediated end-resection of DSBs that is not usually detected in cells with intact telomeres (Fig. 5c). Indeed, the hybridization probe detected C-rich telomeric ssDNA in CRISPR-Cas9 modified 293T cells in which telomeric DSBs had been introduced, but did not detect C-rich telomeric ssDNA in normal 293T cells or in control cells exposed to scrambled sgRNA (Fig. 5d). As expected, the C-rich ssDNA detected by this probe was sensitive to Exonuclease I, an ssDNA-specific 3′-exonuclease. Treatment of DNA with Exonuclease I eliminated the putative HR-intermediate, preventing detection by the strand-specific telomeric hybridization probe (Fig. 5c,e).

As a result of HR in telomeres, it is expected that putative T-SCE will be increased. To determine the frequency of T-SCE in CRISPR-Cas9-modified cells, we performed chromosome orientation-FISH (CO-FISH)^44^. We observed T-SCE in 13.09% of the chromosomes (*n*=5148) in cells with telomeric DSBs, while T-SCE only occurred in 3.43% (*n*=4626) of chromosomes in control cells exposed to scrambled sgRNA (Fig. 5f,g). Interestingly, a significant number (3.83%) of telomeric recombination events appeared to involve only one chromatid (yellow foci) (Supplementary Fig. 7), suggesting that recombination between nonsister telomeres occurs. A similar phenomenon was observed in ALT cells^23^.

### DSBR in telomeric DNA is mediated by HR

**Figure 6:**
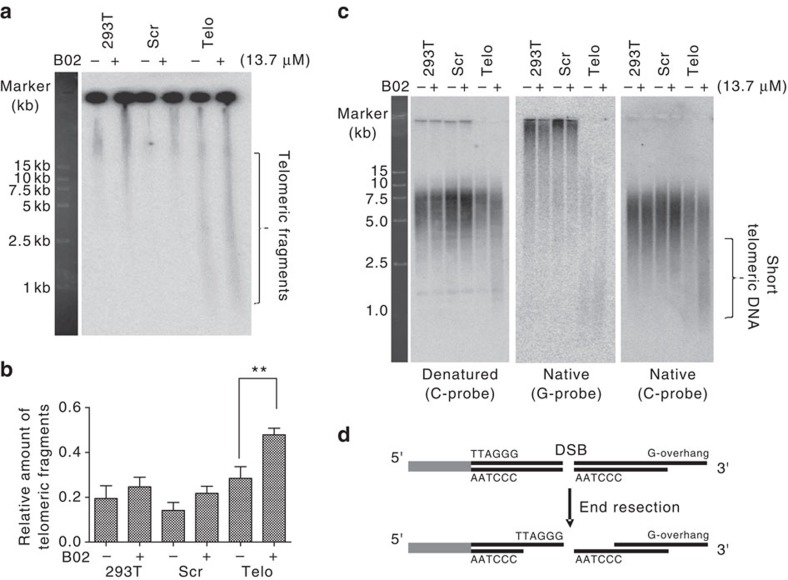
Effect of B02 on telomeric DSBR. (**a**) Cells were treated and analysed as in Fig. 4a, with the addition of B02 (13.7 μM) as indicated. (**b**) Quantification of **a**, values are the average ±s.d. of three independent experiments. The signal intensity was quantified and normalized as described for Fig. 1c. ^**^P<0.01. The Student's *t*-test was used to determine the statistical significance. (**c**) Cells were treated as in **a**. DNA was isolated and analysed using the in-gel hybridization assay. After electrophoresis, telomeric C-probe or G-probe was hybridized under native or denaturing conditions. (**d**) Schematic diagrams show hypothesized repair of DSBs in telomeres.

To confirm the possibility that telomeric DSBR is mediated by HR, we treated CRISPR-Cas9 modified 293T cells with B02, a specific inhibitor of human Rad51 recombinase^45^, and analysed nucleic acids from these cells by CFGE using a telomere-specific probe. After sgRNA-directed introduction of telomeric DSBs, telomeric fragments appeared as a fast-moving smear in the gel, which increased in intensity in the presence of B02 (Fig. 6a,b), indicating that telomeric DSBR was inhibited. Consistently, B02 treatment led to an increase in cells displaying telomeric 53BP1 foci, suggesting that Rad51, and hence HR, is indispensable for telomeric DSB repair (Supplementary Fig. 8a,b). This inhibitory effect was also observed in normal 293T cells and in control cells exposed to scrambled sgRNA, suggesting that endogenous DSBs in telomeres are likely to be repaired by HR (Fig. 6a,b).

To further confirm the function of Rad51 in telomeric DSBR, we initiated Cas9-induced DSBs at telomeres in cells subjected to siRNA mediated depletion of Rad51 (Supplementary Fig. 9a). Similar to the result from B02 treatment, telomeric fragments accumulated to a higher degree in Rad51-deficient cells than that in control cells (Supplementary Fig. 9b,c), demonstrating that Rad51 is required for the repair of telomeric DSBs.

We then explored how the inhibition of telomeric DSBR affects telomere length. Using the TRF assay we found that CRISPR-Cas9-modified 293T cells have the same telomere length as normal 293T cells or control cells exposed to scrambled sgRNA (Fig. 6c, left panel). B02 treatment resulted in decreased telomere length and increased abundance of short telomere-homologous dsDNA fragments in CRISPR-Cas9-modified 293T cells (Fig. 6c, left panel). In-gel hybridization with the G-probe or C-probe under native conditions showed that these short telomeric fragments consist of both C-rich and G-rich telomeric ssDNA (Fig. 6c, middle and right panel). These results support the hypothesis that B02 inhibits telomeric DSBR, thereby resulting in the accumulation of unrepaired broken telomeres (that is, broken telomeres that were processed by HR to have 3′ single-stranded G-rich DNA and the released telomeric fragments that carry a single-stranded C-rich DNA and G-rich overhang) (Fig. 6d, bottom panel).

## Discussion

Whether DNA damage that occurs in telomeres is sufficiently accessible for efficient repair by DNA repair enzymes has been a matter of debate. However, the idea that telomeric DNA damage persists in the typical eukaryotic cell such as a stem cell is improbable, because telomeric DNA damage would often result in the death of the cell, which could have serious consequences at higher levels of the organism. Consistent with this view, our results demonstrate that efficient repair of telomeric DSBs does occur in proliferating cells including normal human fibroblasts and HeLa cells. On the other hand, our results confirm the idea that telomeric DSBs are resistant to repair in cells that senesce due to genotoxic stress.

HR in telomeric DNA is suppressed in normal human cells and in telomerase-positive cancer cells^22^, but it is well recognized that HR is required for telomerase-independent telomere extension in human ALT cells^46^, and that it plays a role at telomeres during early cleavage of embryos^47^. Other evidence supports HR in telomeric DNA, including the presence of HR-related proteins in telomere-specific structures in telomerase-positive cells^24^. In addition, Cho *et al.*^23^ demonstrated that telomeric DSBs promote homology-directed telomere synthesis in ALT cells.

The novel finding of our study is the evidence for HR-mediated DSBR at telomeres in human cells. The data supporting this finding are: (1) telomeres form physically proximate clusters in response to the induction of telomeric DSBs (Fig. 5a,b) and 53BP1 foci preferentially localize to large/clustered telomere spots (Fig. 4b), indicating that HR may occur at the damaged telomeres; (2) detection of DSB-induced telomere-homologous DNA fragments with 3′ C-rich terminal ssDNA, a likely product of end-resection during HR-mediated DSBR (Fig. 5c,d and Fig. 6c,d); (3) increased frequency of T-SCE after induction of telomeric DSBs (Fig. 5f,g); and (4) inhibition of telomeric DSB repair by knockdown of Rad51 or by exposure to B02, a specific inhibitor of HR (Fig. 6 and Supplementary Fig. 9).

Telomeric HR is active in ALT cells, but is generally suppressed in normal and telomerase-positive cells. Coincidently, telomeric chromatin is typically less compact in ALT cells than in telomerase-positive cells^48^. This is consistent with the idea that heterochromatization of telomeric DNA makes it less accessible to DNA modifying proteins, nucleases^49^,^50^ and other enzymes required for efficient HR. In a similar manner, torsional stress is relieved when DSBs are induced in chromosomal DNA^51^,^52^, and it is possible that the associated relaxation of chromatin structure could facilitate HR-mediated DSBR in telomeric DNA.

TRF2 is a component of the telomere-associated shelterin complex, and it is well established that TRF2 inhibits NHEJ in telomeres^11^. This suggests that telomeric DSBR is likely to be carried out in an HR-dependent manner. Consistently, we observed T-SCE in cells with induced telomeric DSBs. Given the observation of clustered telomeres and the fact that highly repetitive telomeric DNA on all chromosome termini provide a unique opportunity for HR, we speculate that the T-SCE may result from recombination between sister and nonsister telomeres. This raises the question of whether HR-mediated repair of telomeric DSBs is only active in late S and G2 or whether it is tightly regulated during the cell cycle, and is an intriguing topic for future study.

Because the subtelomeric region is larger than the typical telomere, it is proportionately more susceptible to DNA damage. We find that small deletions are commonly introduced during DSBR in subtelomeric DNA (Fig. 3f), suggesting that classic NHEJ (C-NHEJ) mediates the repair process. This differs from the result of an earlier study, which concluded that DSBs in subtelomeric DNA sequences are likely to be repaired by alternative NHEJ (A-NHEJ)^53^, leading to large deletions surrounding the DSB. One explanation for the different results of the two studies is the use of different methods to induce subtelomeric DSBs: in the earlier study, DSBs were introduced into a cloned/foreign DSB cleavage site by I-*SceI*, whereas in our current study, DSBs were introduced by Cas9 at an endogenous subtelomeric DNA sequence. However, it is possible that large deletions occurred under our experimental conditions that could not be detected because they were more than 0.5 kb upstream or downstream of the DSB repair site (the limit of the sequenced region) (Fig. 3f). Nevertheless, the results support the conclusion that subtelomeric DSBR is mediated by NHEJ.

It is also important to note that Miller *et al.*^53^ found a similar HR frequency in bulk genomic DNA and in subtelomeric DNA, indicating that HR is not suppressed at subtelomeric regions. Therefore, although our evidence suggests that the majority of subtelomeric DSBs are repaired by NHEJ, HR may be involved in repairing some DSBs, especially those occurring during S and G2.

Proliferating cells normally accumulate a limited number of DNA lesions per cell cycle that are quickly repaired and have no impact on cell proliferative capacity or cell viability. However, if a large number of DNA lesions accumulate in a given cell, the capacity for repair may be exceeded. In this case, some lesions may go unrepaired, and there is a chance that the cell will undergo stress-induced senescence. Consistent with this model, human fibroblasts irradiated with 10 Gy display persistent telomeric DDR and senesce due to genotoxic stress^14^,^33^. Similarly, a prolonged exposure to a high dose of zeocin had the effect of inducing senescence, a state that allowed for the slow repair of DNA lesions in bulk chromatin, but was incompatible with the repair of telomeric DNA damage (Fig. 2b,c).

Senescent cells can repair endogenous and exogenously induced DSBs, indicating that their DNA repair machinery is largely functional^54^. Our results also demonstrate that DSBR in bulk genomic DNA can occur in cells that senesce due to genotoxic stress or replicative exhaustion (Fig. 2b,e). However, DSBR in bulk genomic DNA is more efficient in cells that senesce due to replicative exhaustion (compare Fig. 2b,e), and telomeres in these cells appear to be resistant to zeocin-induced DDR (compare Fig. 2c,f). These observations may reflect the fact that the cells that enter a state of replicative senescence acquire senescence-associated heterochromatin foci^55^,^56^, regions that are likely to be less accessible to DNA modifying proteins and nucleases than normal chromatin^49^,^50^. Thus, it is possible that increased heterochromatization of telomeric DNA suppresses DDR under conditions of extreme stress and/or high genotoxic load. This is an interesting topic for further study.

## Methods

### Cell culture and transfection

293T, HeLa and BJ fibroblast cells were obtained from American Type Culture Collection (Manassas, VA). All cell lines were negative for mycoplasma contamination. HeLa cells were grown in DMEM (Hyclone) supplemented with 10% newborn calf serum (PAA) and 100 U per ml penicillin/streptomycin (Hyclone), fibroblasts (BJ) and 293T were grown in DMEM (Hyclone) with 10% fetal bovine serum (GIBICO) and 100 U per ml penicillin/streptomycin (Hyclone). Cells were cultured at 37 °C and 5% CO_2_. CRISPR plasmid DNA was transiently transfected into 293T cells using the PEI method: plasmid DNA was incubated with PEI for 20 min, added to cells at appropriate confluence (50–60%) and incubated for 6 h. The medium was exchanged for fresh medium, and cells were incubated for 24 h. Rad51 was knocked down by transient transfection of siRNA (si-1: 5′-GGAAGAAGCUGGAUUCCAUTT-3′; si-2: 5′-GCAACUGAAUUCCACCAAATT-3′; negative control: 5′-UUCUCCGAACGUGUCACGUTT-3′.

### Plasmid construction

The lenti-CRISPRv2 consisting of Flag-Cas9 enzyme and sgRNA was purchased from Addgene (#52961) (ref. 57). To improve the stability and affinity, the scaffold sequence of sgRNA was modified to 5′-NNNNNNNNNNNNNNNNNNGUUUAAGAGCUAUGC UGGAAACAGCAUAGCAAGUUUAAAUAAGGCUAGUCCGUUAUCAACUUGAAAAAGUGGCACCGAGUCGGUGCUUUUUUU-3′ (ref. 40). To induce DSBs in telomeres, the guiding sequence of GTTAGGGTTAGGGTTAGGGTTA (referred to as ‘Telo' in the text and figures) was cloned into lenti-CRISPRv2 (ref. 40). Scrambled sequence (TGCTCCGTGCATCTGGCATC, referred to as ‘Scr' in the text and figures) was used as a control. To induce DSB at the subtelomeric region of Xp-Yp chromosomes, the guiding sequences (CCTAAATCCCAGATGGGAAC and GCACGTGGAAG AAGCTATCG for induction of DSB at 0.5k and 1.0k, respectively) were cloned into lenti-CRISPRv2 and transfected into 293T cells.

### T7E1 assay and TA cloning

PCR was performed to amplify the target sequences (PCR primers for 0.5 kb: forward-CCCTCTGAAAGTGGACCTATCAG; reverse-TGGGGATATGACTGCT CCCTTT; PCR primers for 1.0 kb: forward-CACTAGGACCCTGAGACAAC; reverse-CATACTCGGAAGGACAATC); amplicons were then denatured and reannealed slowly to generate heteroduplexes; the reannealed products were cleaved by T7 endonuclease I (T7E1) and resolved on a 2% agarose gel^58^. For TA cloning and sequencing, amplicons were cloned into the TA vector according to the manufacturer's instructions (TA-cloning Kit, TAKARA). TA clones were sequenced using the universal TA-sequencing primer (IGE Biotech).

### Western blot

Cells were lysed in lysis buffer and boiled for 15 min. Proteins were separated by SDS–polyacrylamide gel electrophoresis, transferred to polyvinylidene difluoride membrane, and probed with antibodies specific for Flag (1:1,000 dilution, F1804, Sigma) or Rad51 (1:500 dilution, sc-8349, Santa Cruz). GAPDH (1:5,000 dilution, 60004-1-Ig, proteintech) or β-actin (1:2,000 dilution, 66009-1-Ig, Proteintech) antibodies were used to determine protein amounts as a loading control. Uncropped blots were provided in Supplementary Fig. 10.

### Immunofluorescence-FISH

Cells were grown on a coverslip, fixed in 4% paraformaldehyde for 15 min at room temperature, followed by incubation at room temperature for 30 min in 0.5% Triton X-100. The coverslip was washed and incubated with blocking solution for 1 h at room temperature. Primary antibody (1:2,000 dilution, 53BP1, NB100-304, Novus Biologicals) in blocking solution was added, cells were incubated overnight at 4 °C, washed with blocking solution, and then incubated with blocking solution containing DyLight 488 or 549 conjugated secondary antibody for 1 h at room temperature. The coverslip was washed, incubated in 4% paraformaldehyde for 10 min, washed in ethanol series solutions, denatured at 85 °C for 3–5 min, hybridized with PNA probe (Panagene) for 2 h at 37 °C, washed and mounted with DAPI stain and visualized using a Zeiss microscope. Antibodies/probes: rabbit polyclonal anti-53BP1 antibody (Novus), Cy3-labelled CCCTAA, PNA probe (Panagene, Korea).

### Chromosome orientation FISH

After transfection with plasmid for 24 h, cells were incubated with BrdU for 14 h, with addition of nocodazole (0.5 μg ml^−1^) after 11 h. Cells were collected, transferred to 0.075 M KCl, incubated for 30 min at 37 °C, recovered and washed three times with methanol:acetic acid (3:1). Cells were transferred to slides, digested with pepsin (1 mg ml^−1^) for 40 s, treated with ultraviolet (365 nm, UVP-CL1000) in the presence of Hoechst for 35 min. Samples were treated with EXO III (100 U for 2 h at 37 °C), hybridized with TelC and TelG, mounted with DAPI and observed using a Zeiss microscope^59^.

### Constant-field gel electrophoresis

Cells were imbedded in 0.7% agarose, lysed with 0.5% SDS in Tris-HCl and digested with RNase A (100 μg ml^−1^) and proteinase K (250 μg ml^−1^) at 37 °C overnight^30^. Gel electrophoresis was performed using 0.7% agarose in TAE buffer. In-gel hybridization analysis of telomeric DNA was performed as follows^60^: the gel was dried for 1 h at room temperature and hybridized overnight at 42 °C with a telomere probe in 1 × hybridization buffer (2 μg ml^−1^ sonicated Escherichia coli DNA, 10 × Denhards' buffer, 0.5% SDS and 5 × SSC). The gel was washed four times in wash buffer (2 × SSC, 0.5% SDS) and exposed to a PhosphorImager screen (GE Healthcare Life Science). Blots were quantified and the fraction of telomeric fragments was calculated, defined as ‘(the intensity of signal of telomeric fragments)/(total intensity of signal in whole lane)'. Uncropped gels can be found in Supplementary Fig. 10.

### 2D agarose gel electrophoresis and hybridization

Briefly, 10 μg of genomic DNA was digested with RsaI and HinfI (Fermentas, Thermo Scientific) and loaded onto a 0.4% agarose gel^61^. Electrophoresis was carried out in 1 × TBE at 1 V cm^−1^ for 12 h at room temperature. The lane containing DNA was excised from the gel and the gel buffer was exchanged with 1 × TBE with 0.3 μg ml^−1^ ethidium bromide (EB) (Sigma). The gel slice was placed and cast with 1% agarose gel in 1 × TBE containing 0.3 μg ml^−1^ EB. The gel was run at 4 °C for 6 h at 3 V cm^−1^ (refs 62, 63). The hybridization was performed as above. For the ExoI experiment, genomic DNA was treated with 20 units of Exo I (NEB) for 2 h prior to 2D gel electrophoresis.

### SA-β-gal staining and cell apoptosis assay

The senescence-associated beta-galactosidase (SA-β-gal) staining assay was performed using an SA-β-gal staining kit (Sigma) and by following the manufacturer's instructions. The cell apoptosis assay was performed using the Annexin V/PI apoptosis Kit (Sigma) and by following the manufacturer's instructions.

### Ionizing radiation

Exposure of cells to ionizing radiation was accomplished using an X-Ray Biological Irradiator (RS2000, Rad Source Technologies). The irradiation dose rate was 1.2 Gy min^−1^ for human BJ fibroblast cells.

### In-gel telomere overhang assay

Briefly, isolated genomic DNA was digested with Rsal1, Hinf1 and Msp1 (Thermo Scientific) and resolved on a 0.7% agarose gel. The gel was dried at room temperature and hybridized with telomeric C-probe or G-probe^64^. The single-stranded overhang signal was obtained by exposing the gel to a Phosphoimager screen. Afterwards, the gel was denatured in alkali solution (0.5 M NaOH, 1.5 M NaCl), neutralized (0.5 M Tris-HCl, pH 8.0, 1.5 M NaCl) and re-hybridized with telomere probe.

### Statistical analyses

The Student's two-tailed unpaired *t*-test was used to determine the statistical significance, and the resulting *P* values are indicated in the figures (*P<0.05; ^**^P<0.01; ^***^P<0.001). Sample size was chosen as previously described^65^. No randomization of samples was performed, and no blinding was done. The data meet the assumptions of the tests and statistical tests are justified as appropriate. The variances between the groups are similar, which are statistically compared. None of the samples is excluded.

### Data availability

All the data that support the findings of this work are available from corresponding authors on request.

## Additional information

**How to cite this article:** Mao, P. *et al.* Homologous recombination-dependent repair of telomeric DSBs in proliferating human cells. *Nat. Commun.* 7:12154 doi: 10.1038/ncomms12154 (2016).

## Supplementary Material

Supplementary Information Supplementary Figures 1-10
