# Supplementary material for: Homologous recombination-dependent repair of telomeric DSBs in proliferating human cells
Source: Nat Commun. 2016 Jul 11;7:12154. doi: 10.1038/ncomms12154 (PMC4942568; doi:10.1038/ncomms12154)
Supplement: Supplementary Information — Supplementary Figures 1-10 [file ncomms12154-s1.pdf]

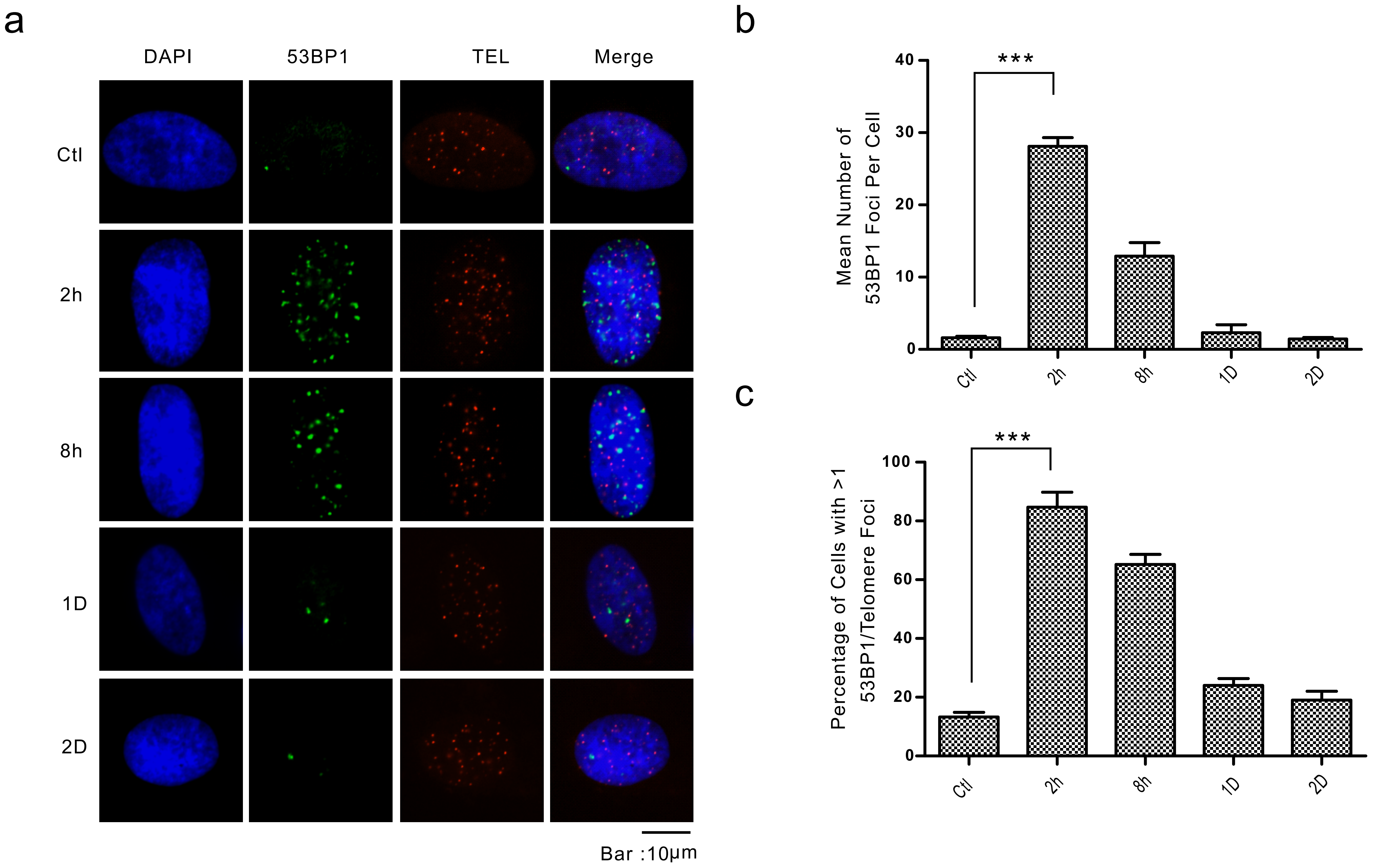

Supplementary Figure1:Repair of telomeric DSBs induced by ionizing irradiation in BJ fibroblast cell.(a) Cells were exposed to ionizing irradiation(2Gy-X ray) and released for 2,8h,1day and 2days.Untreated cells served as a control(Ctl).DDR and telomeres were visualized by antibody against 53BP1 and probe to telomeric sequence, respectively. Scale car:10 μm. (b) Quantification of a. Mean number of 53BP1 foci per cell was determined. (c)Quantification of a. The percentage of cells with 1 or more telomeric 53BP1foci per cell was calculated. All values are average ± SD of three independent experiments(n≧100). ***p<0.001,the student's t-test was used to determine the statistical significance.

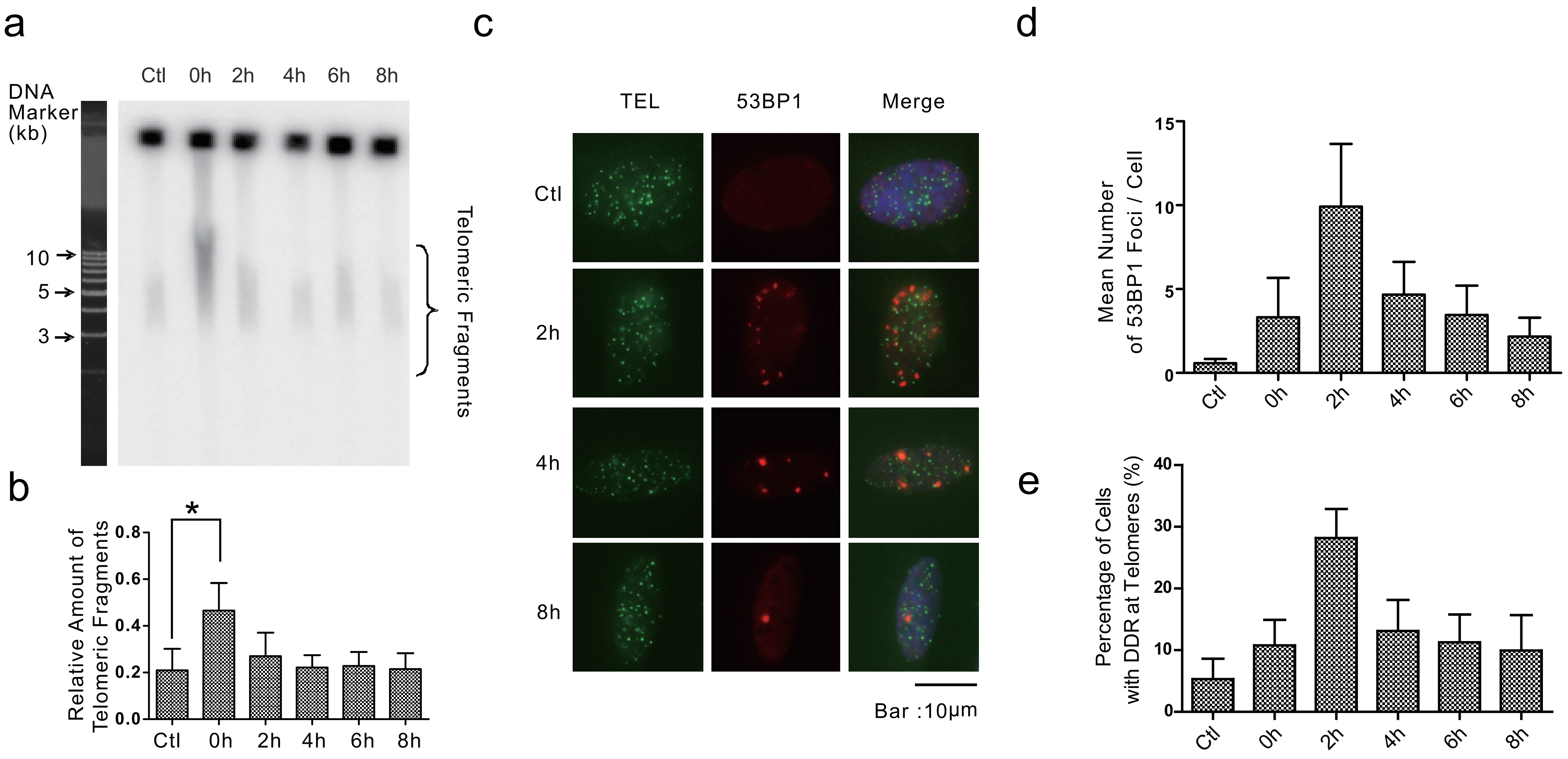

Supplementary Figure 2:Repair of telomeric DSBs in HeLa cells.(a)cells were exposed to zeocin (100μg per ml) for 1h and released to fresh medium for 0,2,4,6 and 8h. Cells harvested at the indicated time points were assayed for determination of telomeric fragments by constant field gel electrophoresis(CFGE).Corresponding MW was indicated on the left.(b)Quantification of a. Relative amount of telomeric fragments represent the signal intensity normalized to intensity in the entire sample. Untreated cells served as a control(Ctl).*p<0.05.(c)Cells was exposed to zeocin (100μg per ml) for 1h and released to fresh medium for 0,2,4, 6,8h. Untreated cells served as a control(Ctl). DDR and telomeres were visualized by antibody against 53BP1 and probe to telomeric sequence, respectively.Scale bar:10μm.(d)Quantification of c. Mean number of 53BP1 foci per cell was determined.(e)Quantification of c. The percentage of cells with 1 or more telomeric 53BP1 foci per cell was calculated. All values are average ± SD of three independent experiments(n≧100).The student's t-test was used to determine the statistical significance.

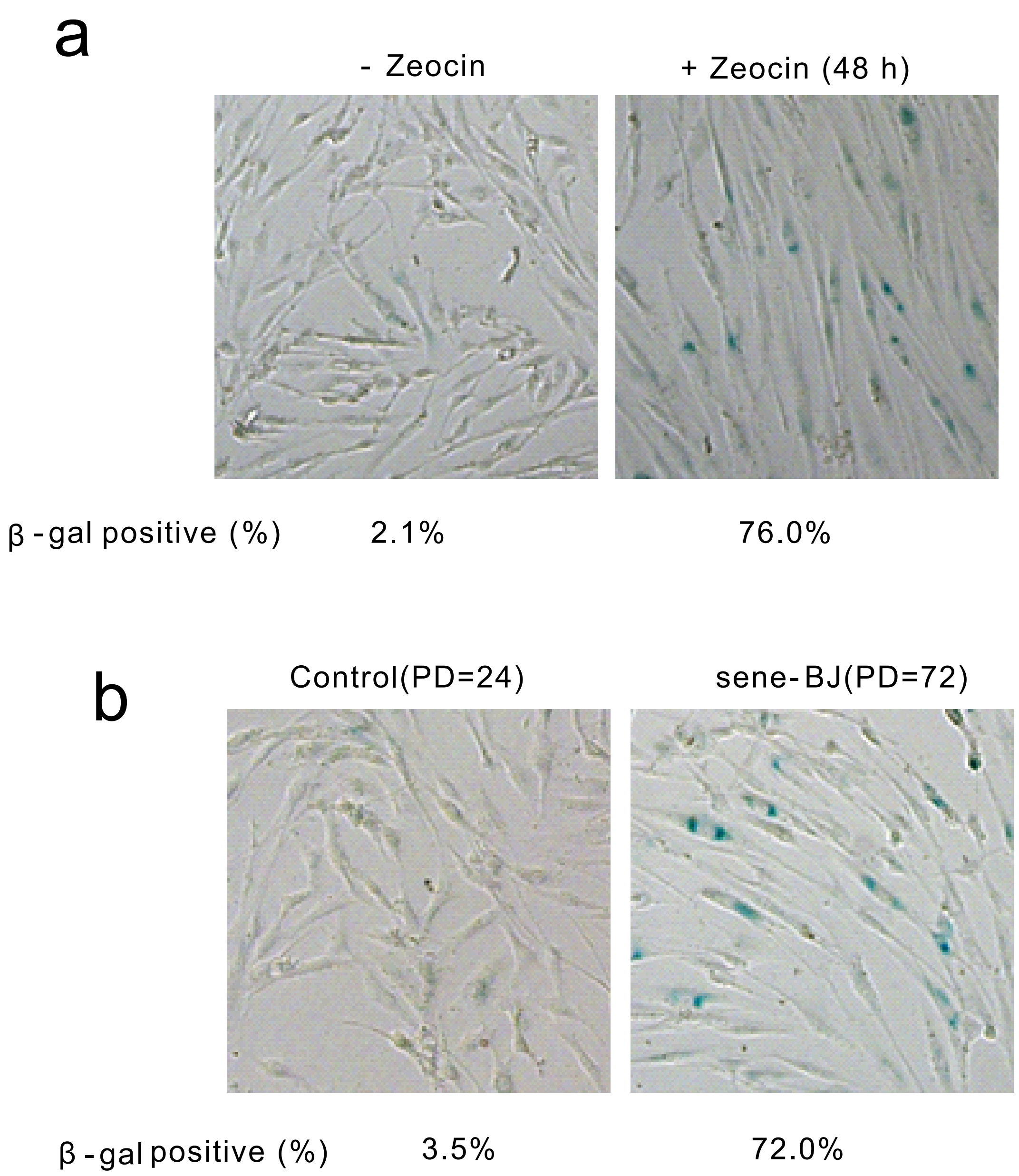

Supplementary Figure 3: Stress induced senescent and replicatively senescent cells were verified by SA-β-gal staining (a)Cells treated with 100μg per ml zeocin for 48h were assayed for β-gal staining.Untreated cells (-Zeocin) served as a control.(b)Young(PD=24) and old fibroblast cells (PD=72) were assayed for β-gal staining. The β-gal positive cells were calculated by counting more than 1000 cells.

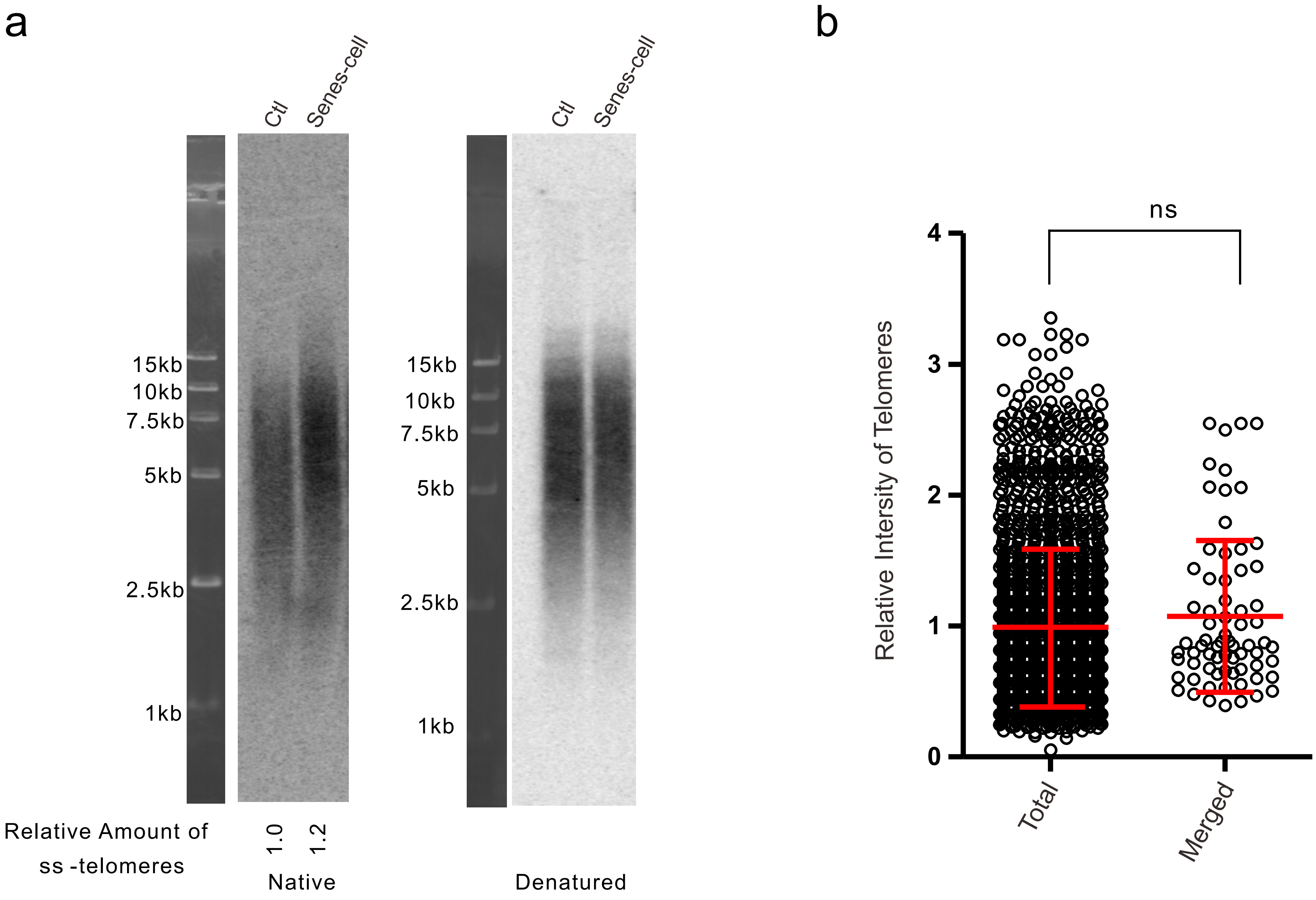

Supplementary Figure 4: Telomere uncapping is not observed in stress-induced senescent BJ fibroblast cells(Senes-cell). (a) Native and denatured TRF assay showing no derease in single-stranded telomere signal(ss-telomeres) and no telomere fusion(high MW teloemres). (b) Quantification of relative telomere length in Figure 2a for total telomeres(Total) and telomeres colocalized with 53BP1 foci (Merged). Relative telomere length was determined based on the intensity of FISH. The student's t-test was used to determine the statistical significance .

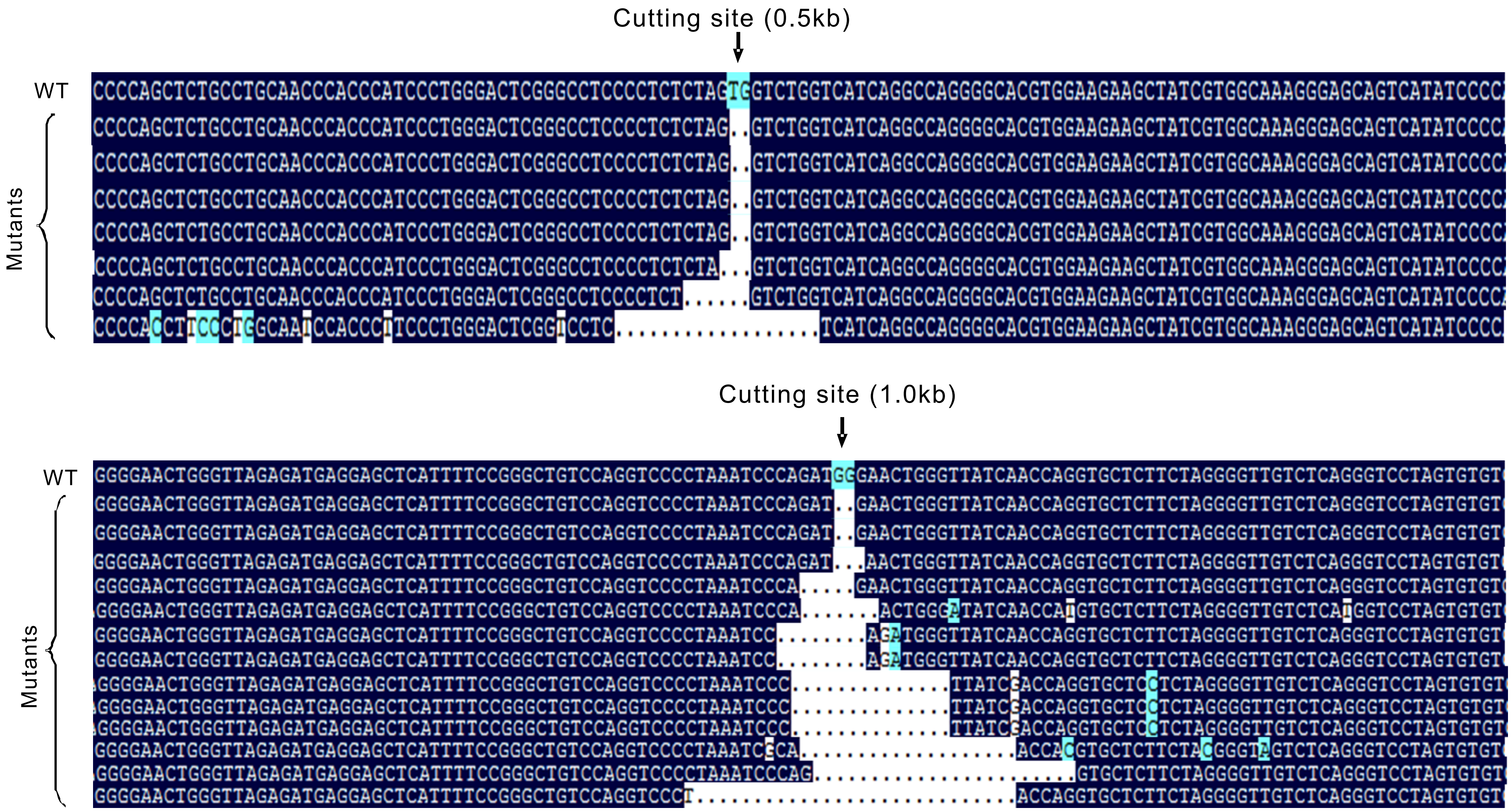

Supplementary Figure 5: Alignments of wild-type(WT) sequence and mutants induced by CRISPR-Cas9 that induce DSBs at DNA 0.5kb or 1.0kb from the first TTAGGG sequence at Xp/Yp chromosomes.

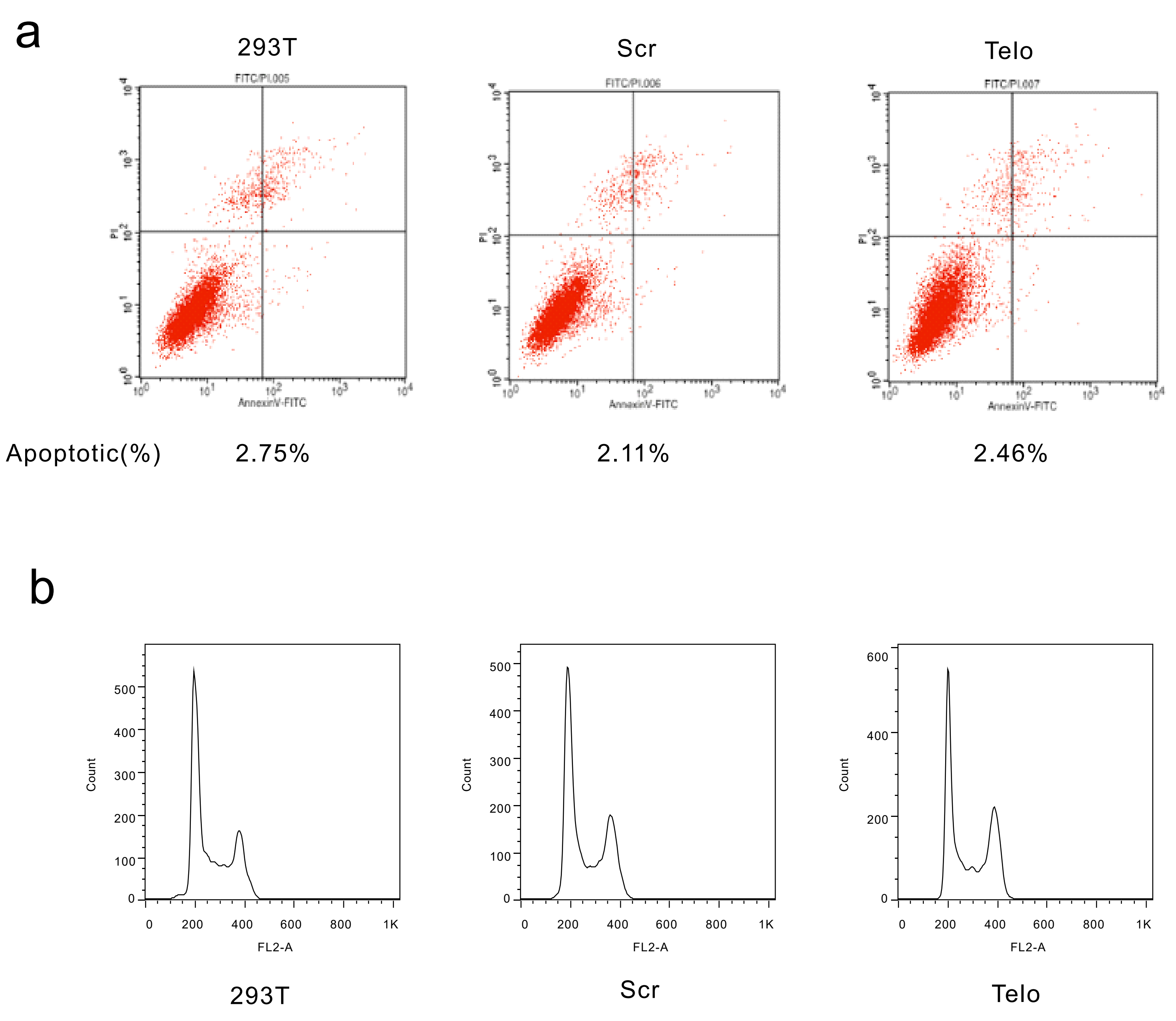

Supplementary Figure 6: CRISPR-Cas9 induced DSBs in telomeres result in neither apoptosis nor cell cycle arrest. (a)FACS analysis of apoptotic cells. (b) FACS analysis of DNA content in 293T cells exposed to telomeric DSBs .Normal 293T cells and cells exposed to scramble sgRNA (Scr) were used as control. cells were assayed 24h after transfection.

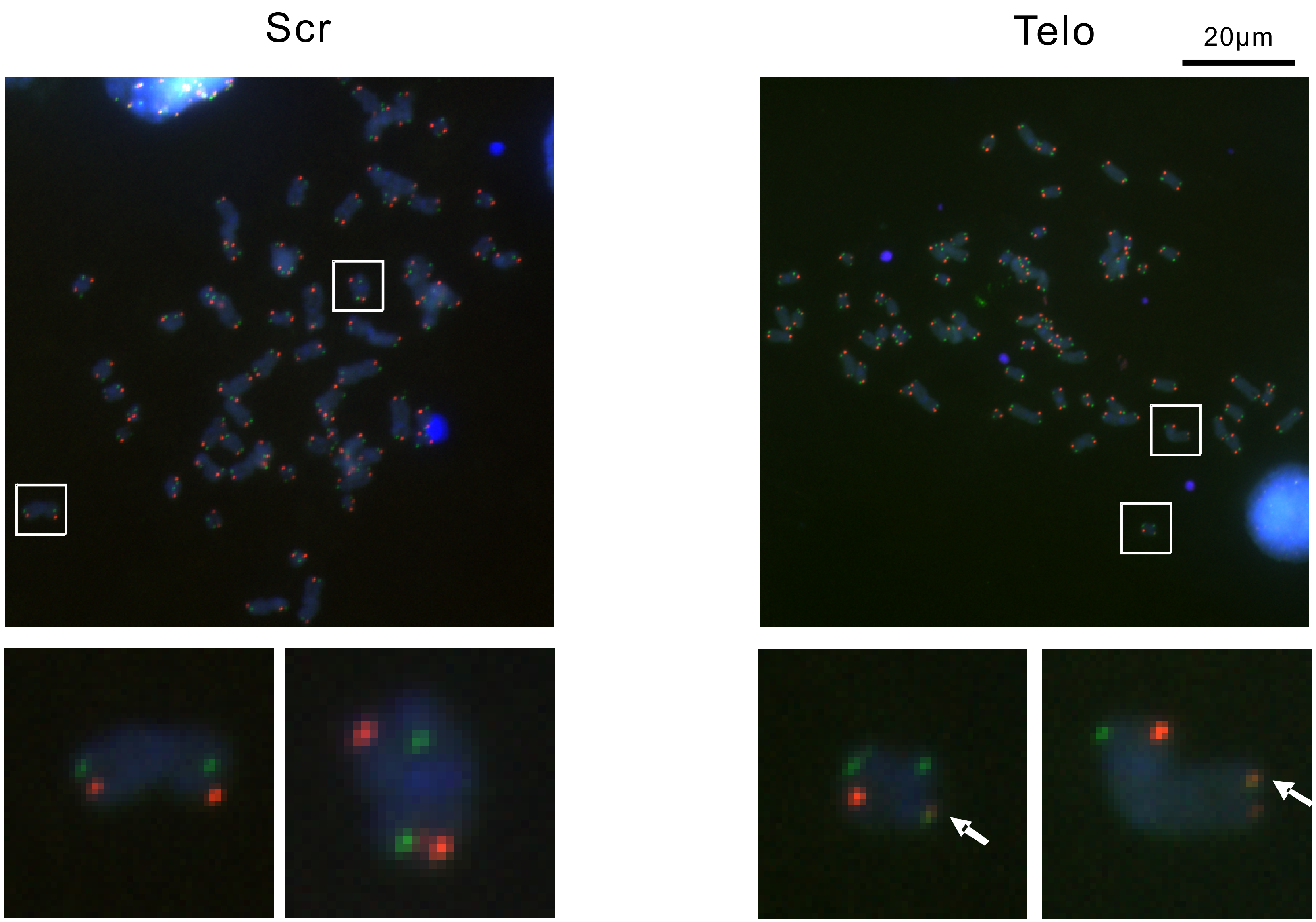

Supplementary Figure 7:Representive images showing HR between telomeres of non-sister chromatids. Telomeric recombination was determined by CO-FISH and recombined telomere indicated by arrow. 293T cells exposed to telomere-targeting sgRNA(Telo) or scramble sgRNA(Scr) were used.Scale bar:20µm.

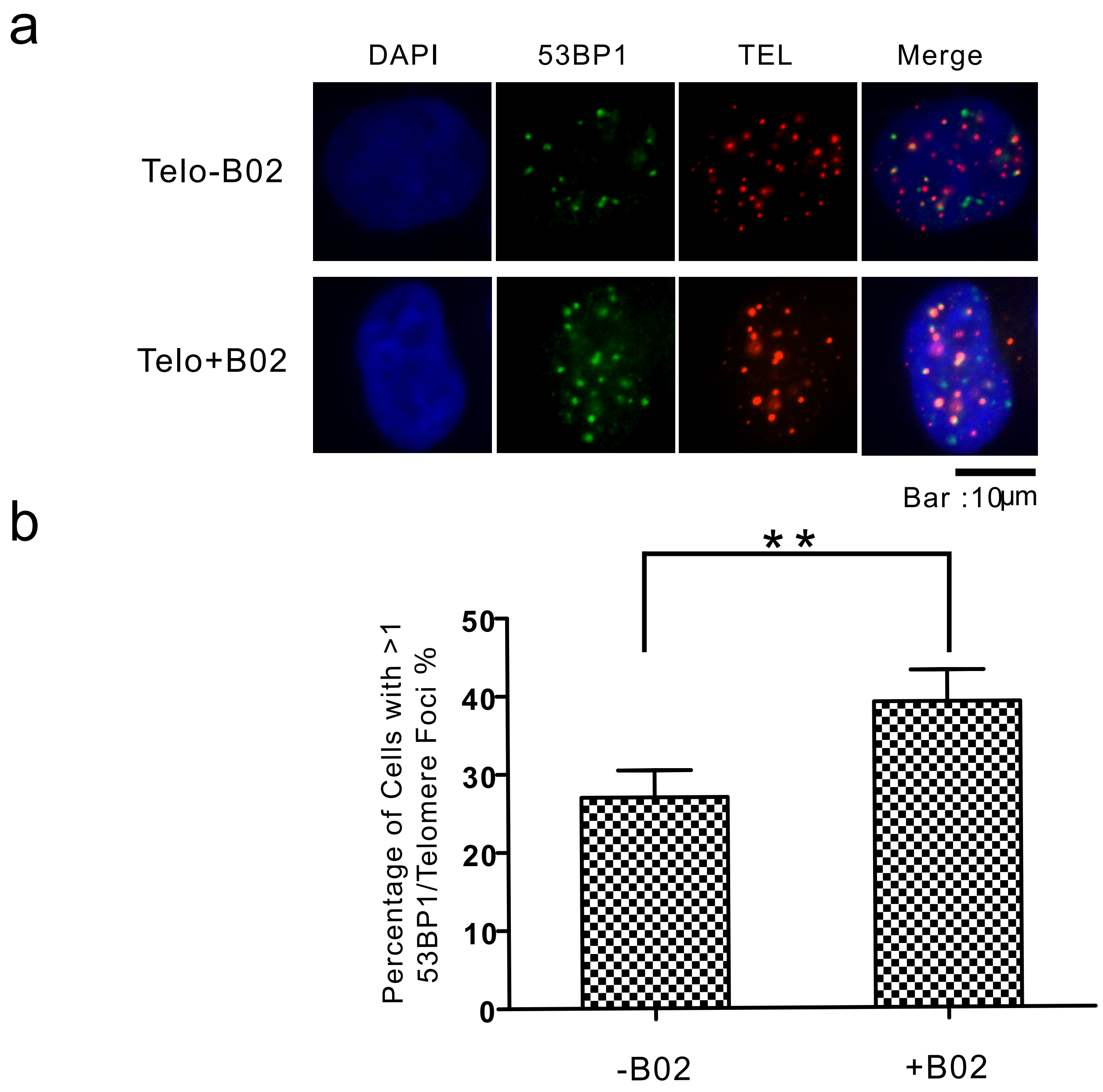

Supplementary Figure8:B02 treatment led to an increase in cells displaying telomeric 53BP1 foci. (a) IF-FISH showing telomeric 53BP1 foci in B02 treated (Cas9 to telomeres and B02:Telo+B02) and untreated (Cas9 to telomeres without B02:Telo-B02) cells. Scale bar:10μm. (b)Quantification of a, data are from three independent experiments(n>50).*P<0.05, the statistical difference was determined by the student's t-test.

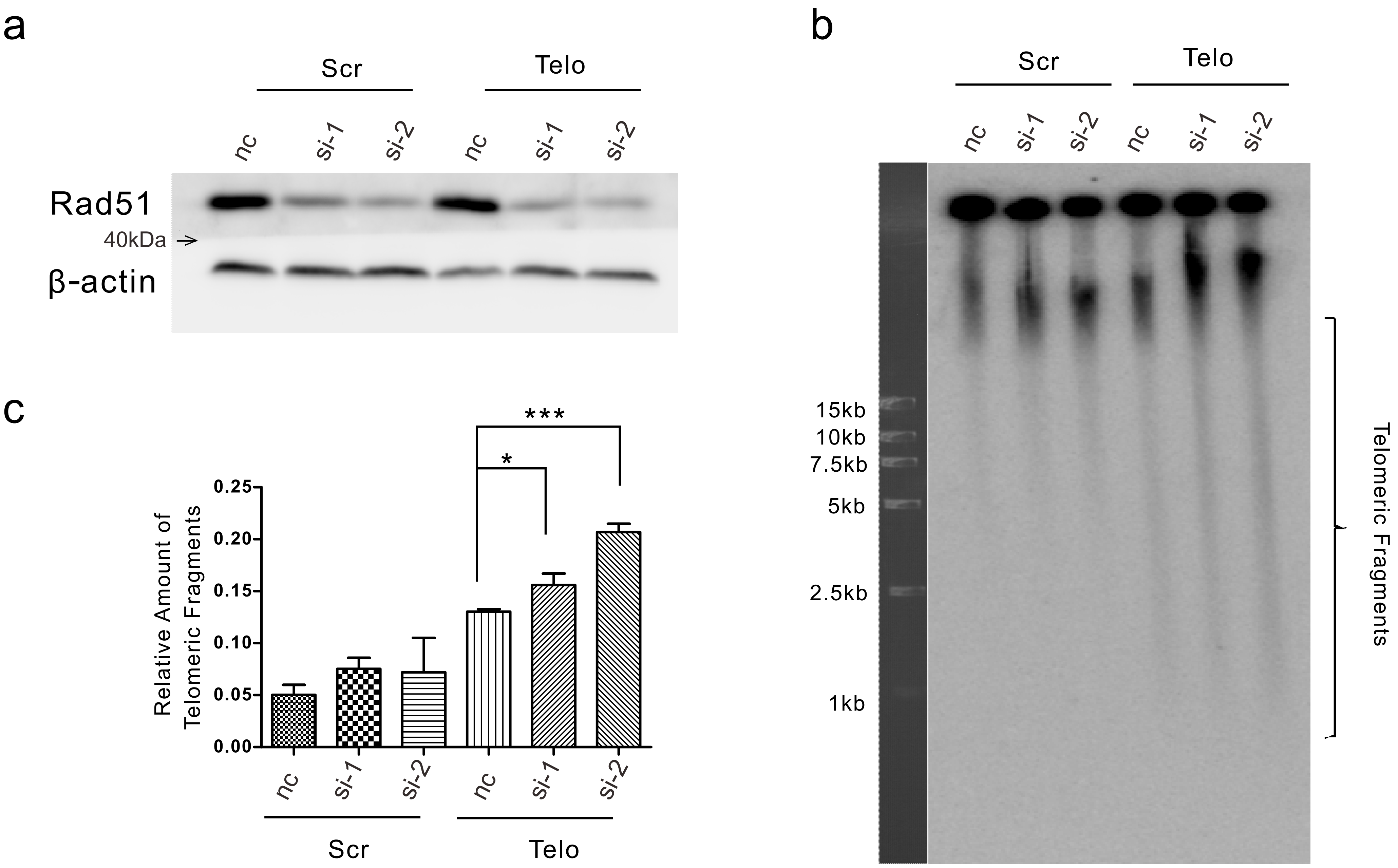

Supplementary Figure 9:Telomeric fragments induced by Cas9-induced telomeric DSBs accumulate in Rad51 deficient cells. (a)Western blot showing the knockdown of Rad51 by siRNA (si-1 and si-2).(b)CFGE and in gel hybridization under native condition showed the accumulation of telomeric fragments induced by CRISPR-Cas9. (c)Quantification of b, data are from three independent experiments. *P<0.05;***P<0.001.The student's t-test was used to determine the statistical significance.

Figure 3b

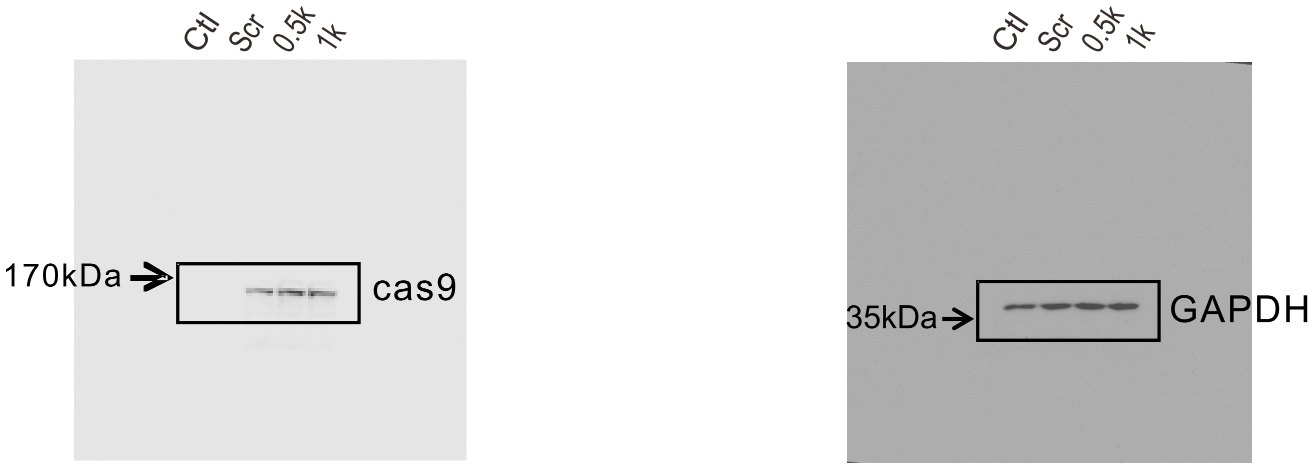

Figure 4a

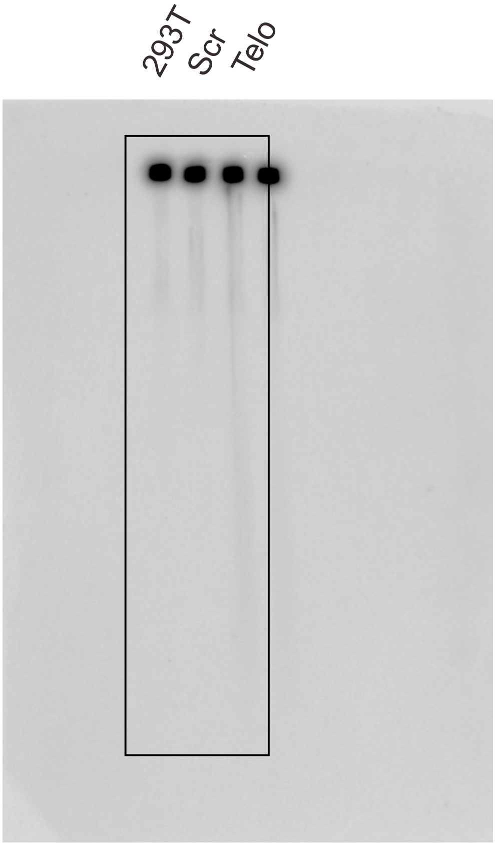

Supplementary Figure 9a

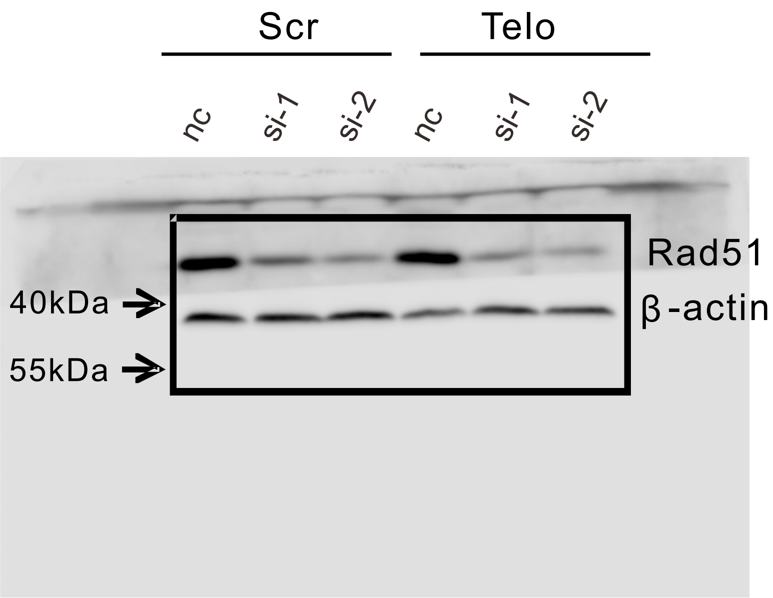

Supplementary Figure 10:The full scans of blots and gel in related figures. Black boxes highlight the indicated lanes in figures.
